# Model-Based Vestibular Afferent Stimulation: Evaluating Selective Electrode Locations and Stimulation Waveform Shapes

**DOI:** 10.3389/fnins.2018.00588

**Published:** 2018-08-30

**Authors:** Peter Schier, Michael Handler, Lejo Johnson Chacko, Anneliese Schrott-Fischer, Karl Fritscher, Rami Saba, Christian Baumgartner, Daniel Baumgarten

**Affiliations:** ^1^Department for Biomedical Computer Science and Mechatronics, Institute of Electrical and Biomedical Engineering, UMIT-Private University for Health Sciences, Medical Informatics and Technology, Hall in Tirol, Austria; ^2^Department of Otolaryngology, Medical University of Innsbruck, Innsbruck, Austria; ^3^Department for Biomedical Computer Science and Mechatronics, Institute of Biomedical Image Analysis, UMIT-Private University for Health Sciences, Medical Informatics and Technology, Hall in Tirol, Austria; ^4^MED-EL GmbH, Innsbruck, Austria; ^5^Faculty of Computer Science and Biomedical Engineering, Institute of Health Care Engineering, Graz University of Technology, Graz, Austria; ^6^Department of Computer Science and Automation, Institute of Biomedical Engineering and Informatics, Technische Universität Ilmenau, Ilmenau, Germany

**Keywords:** vestibular, implant, human, electrical stimulation, selectivity, energy, finite element, fibers

## Abstract

A dysfunctional vestibular system can be a severe detriment to the quality of life of a patient. Recent studies have shown the feasibility for a vestibular implant to restore rotational sensation via electrical stimulation of vestibular ampullary nerves. However, the optimal stimulation site for selective elicitation of the desired nerve is still unknown. We realized a finite element model on the basis of μCT scans of a human inner ear and incorporated naturally distributed, artificial neural trajectories. A well-validated neuron model of myelinated fibers was incorporated to predict nerve responses to electrical stimulation. Several virtual electrodes were placed in locations of interest inside the bony labyrinth (intra-labyrinthine) and inside the temporal bone, near the target nerves (extra-labyrinthine), to determine preferred stimulation sites and electrode insertion depths. We investigated various monopolar and bipolar electrode configurations as well as different pulse waveform shapes for their ability to selectively stimulate the target nerve and for their energy consumption. The selectivity was evaluated with an objective measure of the fiber recruitment. Considerable differences of required energy and achievable selectivity between the configurations were observed. Bipolar, intra-labyrinthine electrodes provided the best selectivities but also consumed the highest amount of energy. Bipolar, extra-labyrinthine configurations did not offer any advantages compared to the monopolar approach. No selective stimulation could be performed with the monopolar, intra-labyrinthine approach. The monopolar, extra-labyrinthine electrodes required the least energy for satisfactory selectivities, making it the most promising approach for functional vestibular implants. Different pulse waveform shapes did not affect the achieved selectivity considerably but shorter pulse durations showed consistently a more selective activation of the target nerves. A cathodic, centered triangular waveform shape was identified as the most energy-efficient of the tested shapes. Based on these simulations we are able to recommend the monopolar, extra-labyrinthine stimulation approach with cathodic, centered triangular pulses as good trade-off between selectivity and energy consumption. Future implant designs could benefit from the findings presented here.

## 1. Introduction

**Figure 1:**
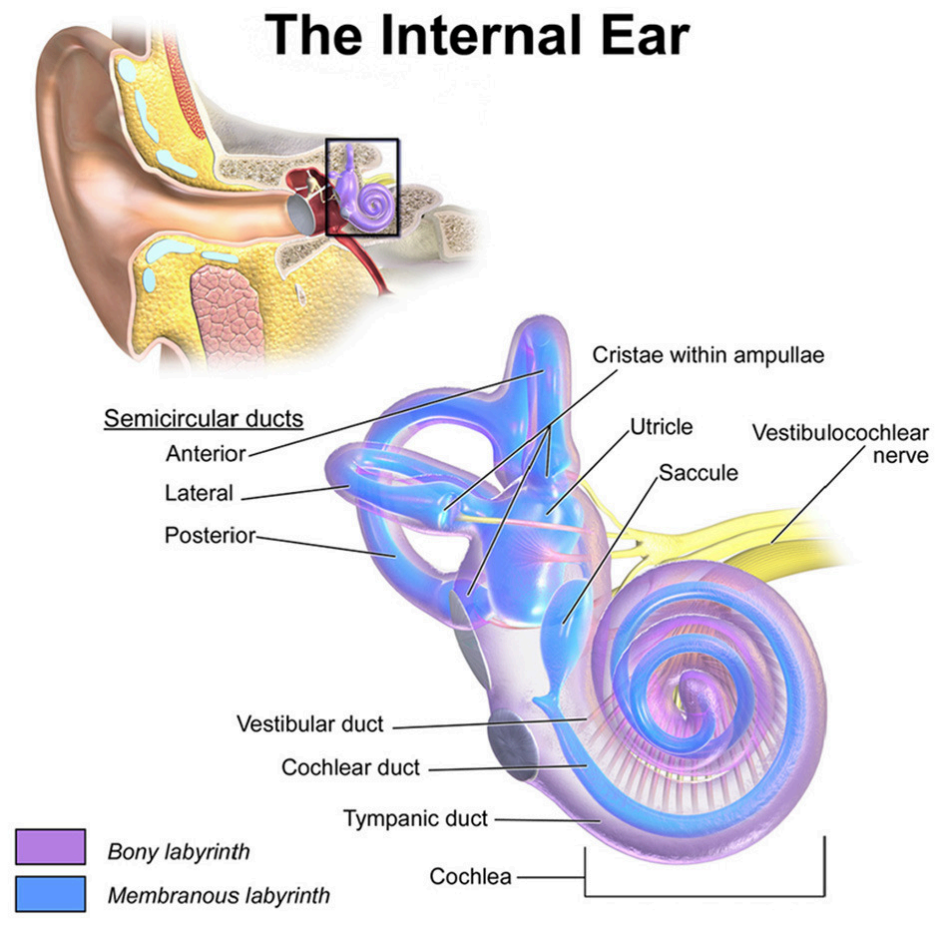
Location and detailed depiction of the human vestibulochochlear system adopted from Blausen.com staff (2014). The anterior, lateral and posterior SCCs sense the rotational accelerations of the head while the two otolith organs, utricle and saccule, encode translational motions. The crista within the ampulla of each SCC is connected to an ampullary nerve, respectively. Together with the utricular, the saccular and the cochlear nerve, they merge into the vestibulochochlear nerve in the inner auditory canal.

The vestibular system has a major contribution to the sense of balance in humans and most other mammals. Accelerations of the head are sensed in the three semicircular canals (SCCs) and the two otolith organs (see Figure 1). Gaze stabilization is another key element of the vestibular system. The vestibulo-ocular reflex links head motion to eye movements and stabilizes images on the retina. Patients with impaired vestibular function due to bilateral vestibulopathy have difficulties maintaining an upright posture and suffer from oscillopsia. The scarcity of therapeutic options and the enormous success of cochlear implants show the necessity of developing a prosthesis to restore defective vestibular sensation.

The feasibility and efficacy of vestibular implants have already been proved in both animals (Della Santina et al., 2007; Fridman and Della Santina, 2012; Rubinstein et al., 2012) and humans (Guyot et al., 2011; Van De Berg et al., 2012; Perez Fornos et al., 2014). In those studies, electrodes were implanted near the anterior, the lateral and/or the posterior ampullary nerves. A partial restoration of the vestibular function via electric stimulation of these nerves has been successfully demonstrated.

**Figure 2:**
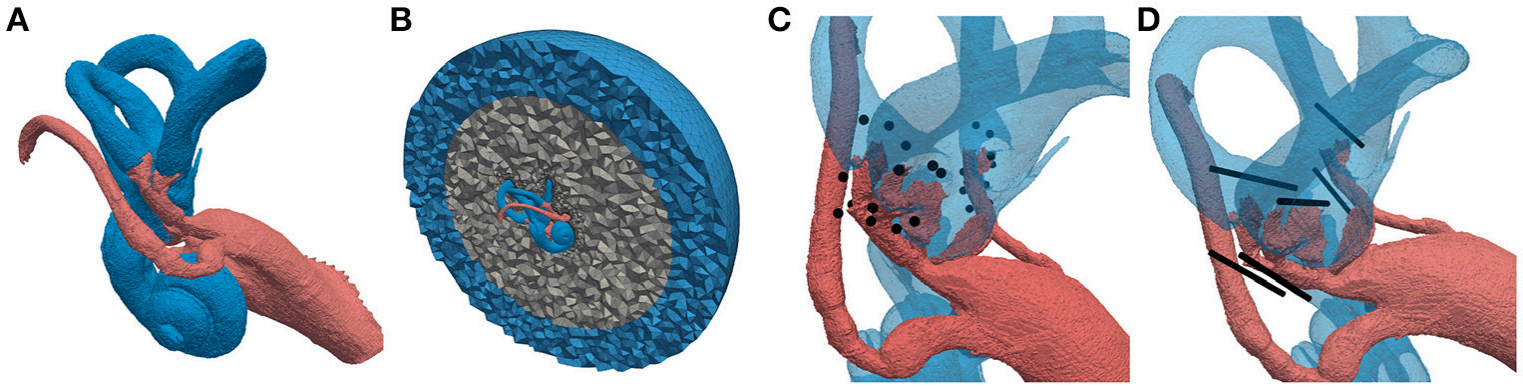
Labeled tetrahedral volume mesh of the vestibular system used for the computation of potential distributions. Different colors of the regions represent different conductivity properties (*red*: nerve tissue, *blue*: lymphatic fluid/saline layer, *gray*: bone, *black*: electrodes). **(A)** Vestibular system including facial nerve and IAC. **(B)** Cross-section of surrounding bone sphere and saline layer. **(C)** Embedded IL and EL, spherical electrodes and **(D)** cylindrical electrode arrays.

Optimal electrode placement during implantation surgery is a difficult task, considering the tiny anatomical structures of the inner ear. Two surgical techniques for the positioning of the electrodes emerged: an intra-labyrinthine (IL) approach, where the electrodes are placed inside the bony labyrinth in the ampullae of the SCCs and an extra-labyrinthine (EL) approach with the electrodes located within the temporal bone in vicinity of the ampullary nerves (see Figures 2C,D). Each surgical approach offers different advantages and disadvantages. The downside of the IL implantation is the increased risk of hearing loss while the EL approach is more complicated to perform (Guyot et al., 2016). A direct comparison is not possible, since no single patient has both IL as well as EL electrodes implanted and the efficacy of the two approaches varies strongly across patients (Guinand et al., 2015). Although both approaches were tested *in vivo*, a clear statement which one enables a more selective elicitation of the target nerves has not been given yet.

Similarly, simulations of vestibular nerve responses to electrical stimulation were performed with IL (Hayden, 2007; Hayden et al., 2011) and EL (Parikh, 2006; Marianelli et al., 2015) approaches. In all of these studies a proper mathematical neuron model has been used to reconstruct the electrophysiological behavior of nerve fibers. Most of them, namely Hayden (2007), Hayden et al. (2011), and Marianelli et al. (2015), incorporated the spatially extended non-linear node (SENN) axon model, which was initially developed by Frijns et al. (1994) for electrical prosthesis design in the cochlea and is based on experimental data (Schwarz and Eikhof, 1987). In the work of Hayden et al. (2011) the model has been modified in order to simulate responses of vestibular nerve fibers in chinchillas during IL stimulation. This included the incorporation of spontaneous discharge regularity (SDR), which is typical for vestibular afferents and essential for the encoding of head motions (Smith and Goldberg, 1986). Marianelli et al. (2015) examined the effects of EL stimulation of human vestibular nerves based on a digitally reconstructed human inner ear. Promising results regarding selective nerve stimulation were shown with both implantation methods. However, each of these studies concentrated on only one of the approaches, respectively. Because of the non-negligible differences between these models, no useful comparison between the efficacy of IL and EL stimulation can be drawn.

Since the exact location, electrode configuration and stimulus waveform for a (near) optimal stimulation of human vestibular ampullary nerves is still unknown, the aim of the present study is to objectively assess and compare different approaches for selective and energy-efficient stimulation of these nerves by means of computer simulations. The goal is to determine a stimulation approach which is clearly preferential in terms of selectivity and energy-consumption. Therefore, in this study we investigate mono- and bipolar configurations of electrodes in IL and EL locations. In addition, we test several different electrode insertion depths for their selectivities to predict possible consequences of misplacement. Also, as there are numerous studies suggesting a considerable influence of pulse waveforms on energy expenditure and charge injection in neural stimulation scenarios (Grill and Mortimer, 1995; Sahin and Tie, 2007; Wongsarnpigoon and Grill, 2010; Wongsarnpigoon et al., 2010) we evaluate several different stimulation pulse shapes with respect to their energy consumption and their nerve excitation efficacy.

## 2. Methods

### 2.1. Overview

A voxel-based 3D image of an excised adult human inner ear was recorded post-mortem via a μCT with contrast enhancement through osmium tetroxide (Johnson Chacko et al., 2018). A high resolution of 15 μm edge length per voxel cube was achieved this way. Bony and membranous labyrinth (see Figure 1), the vestibular nerve with its branches as well as the facial nerve and the inner auditory canal (IAC) were segmented and differently labeled. Because of the excise method the cochlear nerve could not be completely preserved and thus yielded no intact segmentation.

The body was donated to the Division of Clinical and Functional Anatomy of the Innsbruck Medical University by a 70 year old male who had given his informed consent for his use for scientific and educational purposes prior to death (McHanwell et al., 2008; Riederer et al., 2012). The cadaver was preserved using an arterial injection of a formaldehyde-phenol solution/an alcohol-glycerin solution and immersion in phenolic acid in water for one to three month (Platzer et al., 1978).

A principal component analysis of several shapes of vestibular systems showed that the specimen used in this study is close to the “mean shape” of all the vestibular systems that were examined and is therefore considered appropriate for this type of study (Fritscher et al., under review). With this 3D imaging data as foundation, a highly precise tetrahedral mesh of a human inner ear was constructed (Handler et al., 2017). We realized this finite element model (see section 2.2) to compute the potential distribution of stimulation electrodes. The volume conductivities were adopted from measurements of electrical characteristics in animal and human anatomy (Geddes and Baker, 1967; Hayden et al., 2011; Marianelli et al., 2015). A well-validated mathematical vestibular neuron model (Schwarz and Eikhof, 1987; Frijns et al., 1994; Hayden et al., 2011) was adapted to human fiber morphology and incorporated in order to take realistic nerve responses into account (see section 2.3). Variations of electrode configurations (see section 2.4) and stimulation waveform shapes (see section 2.5) were objectively evaluated using a custom selectivity measure (see section 2.6).

### 2.2. Finite element model

#### 2.2.1. Setup description

A tetrahedral volume mesh was produced based on this dataset to enable a computation of the potential distribution Φ, generated by one or more implanted electrodes (see Figure 2A). The underlying label data were incorporated in the volume mesh to emulate realistic fluid, tissue and bone conductivities. Subsequently, the volume mesh was embedded in a bone sphere with a radius of 25 mm representing a simplified surrounding of the vestibular system. Additionally, a saline layer with a thickness of 10 mm surrounding the bone sphere was introduced to ensure proper model boundary conditions as proposed by Marianelli et al. (2015) (see Figure 2B). This was done using a semi-automatic algorithm developed by Handler et al. (2017) which included the mesh tools TetGen (Hang, 2015) as well as CGAL (The CGAL Project, 2016) and resulted in a volume mesh with approximately 18 million elements.

An electrical conductivity value σ was assigned to each tetrahedral element depending on its material label (see Table 1). The peri- and endolymphatic fluids inside the vestibule, the SCCs and the cochlea as well as the surrounding saline layer were assumed to be equally conductive. As nerve tissue is much more conductive along the neural pathways than transverse to them, the nerve volume meshes were modeled with anisotropic conductivities which differentiate between longitudinal and transverse conductivity components. Conductivity values of fluid, bone and nerve tissue were adopted from Hayden et al. (2011) and Marianelli et al. (2015).

**Table 1:** Electrical conductivity values used in the finite element model (Handler et al., 2017).

**Tissue/Material**	***σ* (S m-1)**
Bone	0.0139^a^
Nerve longitudinal	0.3333^a^
Nerve transversal	0.0143^a^
Cochlear nerve	0.1738
Scala tympani/media/vestibuli	2.0^a^
Endolymph/Perilymph	2.0^a^
Saline layer	2.0^b^
Electrode	1.0 × 10^6^

The values were taken from

a Hayden et al. (2011) and

b *Marianelli et al. (2015)*.

*The conductivity of the cochlear nerve was computed using the average of the longitudinal and transversal nerve tissue conductivity since no nerve fibers were generated in this volume*.

Several single spherical electrodes with a diameter of 0.3 mm as well as cylindrical electrode arrays with a diameter of 0.2 mm and a length of 3 mm were placed in IL (inside the SCCs and the vestibule) and EL (inside the temporal bone, near the vestibular nerve branches) positions in the mesh (see Figures 2C,D). All electrode arrays were subdivided into 15 equally sized cylindrical electrodes with diameters of 0.2 mm and lengths of 0.2 mm, respectively.

A large number of electrodes was embedded to ensure the consistency of the mesh throughout all simulations and to avoid the process of re-meshing the dataset for every electrode configuration. Each electrode could either act as current source, which was able to emit anodic and cathodic currents, or as current sink to drain the emitted current. Electrodes assigned neither source nor sink properties were given the same conductivity as the material surrounding these electrodes. Thus, for the computation of the potential distribution, inactive electrodes in the IL and EL space were considered as endolymph/perilymph and bone, respectively. Current sources and sinks were assigned highly conductive material properties with σ = 10^6^ S m^-1^.

#### 2.2.2. Computation of the potential distribution

For realistic stimulation scenarios of the nerve tissue, it was essential to reproduce the natural current distribution of the embedded electrodes. The electric potential distribution was calculated by solving the Poisson equation

(1) $$\begin{array}{r} -\nabla \cdot \left(\sigma \nabla \text{Φ}\right)=\nabla \cdot \vec{j}, \end{array}$$

where Φ is the electric potential and $\vec{j}$ is the current density vector. Each current source was modeled with a constant current density $\vec{j}$, initially emitting a current *I* = 1 A through its surface. The unit amperage was chosen, since the potential distribution is scaled in relation to the stimulus amplitude during the computation of nerve fiber excitation thresholds (see section 2.3.2). The assumption of quasi-static conditions in the vestibular system enabled linear scalability. Every current sink represented an electrical ground with fixed potential Φ = 0 V. The boundary condition at the outer border of the saline sphere Γ depended on the number of electrodes posing as current sinks in the mesh. If no electrode was defined as current sink (monopolar stimulation), a homogeneous Dirichlet boundary

(2) $$\begin{array}{r} \text{Φ}\left(\text{Γ}\right)\approx \Phi \left(\infty \right)=0 \end{array}$$

was imposed to resemble the electrical ground (i.e., the reference electrode) in infinite distance Φ(∞). Otherwise a perfectly insulating homogeneous Neumann boundary

(3) $$\begin{array}{r} \sigma \frac{\partial \text{Φ}\left(\text{Γ}\right)}{\partial \vec{n}}=0, \end{array}$$

where $\vec{n}$ is the normal vector of the boundary Γ, ensured a current flow exclusively from source to sink electrodes (bipolar stimulation).

### 2.3. Neuron model

#### 2.3.1. Neuron morphology

**Figure 3:**
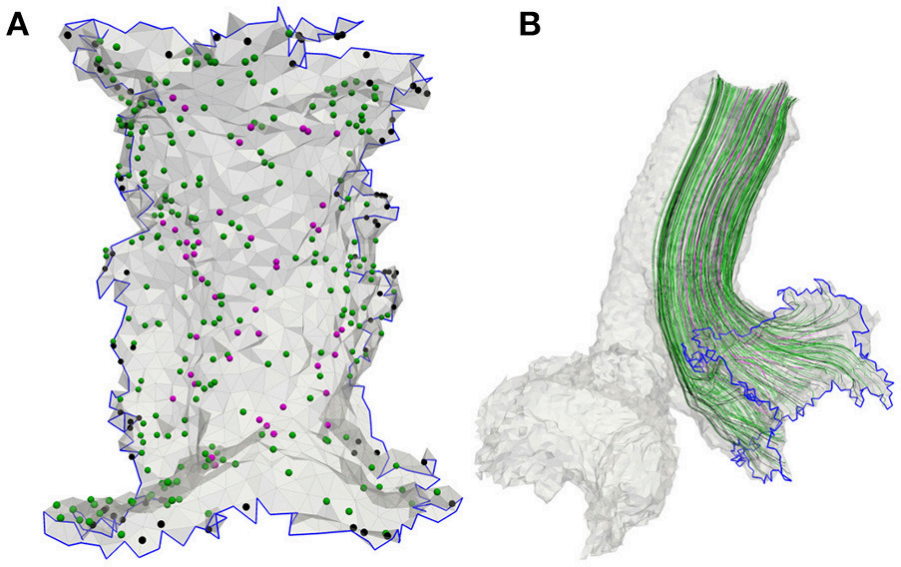
**(A)** Meshed sensory epithelium of the anterior ampullary nerve with 400 fiber start points including calyx (magenta), bouton (black) as well as dimorphic units (green) and **(B)** the corresponding neurons grown through the nerve volume.

Nerve fibers which start in the center are thicker and more excitable than fibers emerging from peripheral regions of the sensory epithelia (Baird et al., 1988). Since excitability strongly depends on the fiber types and their characteristic morphologies, they were considered in the model. The sensory epithelia in the model were subdivided into three equally sized areas: central, peripheral and intermediate (Fernández et al., 1988). The neurons could be distinguished into three fiber types: *calyx units* were located mainly in the central area of the sensory epithelium, *bouton units* occured mostly in the peripheral area and *dimorphic units* were scattered over the entire start-surface (see Figure 3A). Axonal diameters of vestibular nerves were taken from Lopez et al. (2005). Diameters of the facial nerve were taken from Thurner et al. (1993) and since the exact fiber distribution in the IAC was not known, diameters from the results of Lopez et al. (2005) were assumed as well. Axon diameters *d* of the different neurons and their frequencies of occurrence are listed in Table 2. The algorithm by Handler et al. (2017) was used to grow 400 nerve fibers through each nerve volume (see Figure 3B).

**Table 2:** Axonal mean diameters and their standard deviations (STD) are adopted from published data obtained from human specimens.

**Fiber type**	**Axonal diameter** ***d*** **[μm]**		**Occurence (%)**
	**Mean**	**STD**	
Calyx	6.5	±0.5	11.1
Dimorphic	4.0	±0.5	67.4
Bouton	2.5	±0.5	21.5
**Nerve Tissue**			
Facial	5.0	±2.0	
IAC	4.0	±1.3	

*Vestibular ampullary, utricular and saccular nerves are constituted of calyx, dimorphic, and bouton fibers with the stated frequencies of occurence (Fernández et al., 1988; Lopez et al., 2005). Neuron diameters in the facial nerve and the IAC are adopted from Thurner et al. (1993) and Lopez et al. (2005), respectively*.

The internodal distance *L* shows a linear dependency with respect to the myelinated fiber diameter and was approximated for every fiber by the relationship

(4) $$\begin{array}{r} L=\frac{100}{r_{g}}\cdot d \end{array}$$

where *r*_*g*_ = 0.7 is the ratio between the axonal diameter *d* and the diameter of the myelinated fiber (Hursh, 1939; Dodge and Frankenhaeuser, 1959; Hildebrand and Hahn, 1978). The gap width *l* of the nodes of Ranvier was set to 1 μm. Only distal heminodes in vestibular ampullary, utricular and saccular nerves (the first node of every neuron, located at the sensory epithelium) had a larger gap width of *l* = 2 μm (Hayden et al., 2011).

#### 2.3.2. Electrical model

The electrical neuron model used in this work is based on Hayden et al.'s modified SENN model (Hayden et al., 2011). In this model, the myelin sheaths are perfect insulators and transmembrane currents appear exclusively at nodes of Ranvier. These currents are comprised of ion flux through voltage-gated Na^+^- and K^+^-channels as well as currents due to membrane capacitances and leak conductances. Adjacent nodes of Ranvier in a neuron are interconnected via axoplasmic conductances. The previously computed potential distribution was scaled according to the stimulus amplitude of the investigated protocols, linearly interpolated at each node of Ranvier and served as input for the neuron model as extracellular potential.

The incorporated SDR encoded head movements by depolarizing vestibular afferents in variable frequencies depending on movement direction and acceleration. Baseline depolarization rate in the unmoving head was around 100 Hz. The SDR varied among different fiber types. It was classified via the normalized coefficient of variation CV^*^ (Goldberg et al., 1984), corresponding to the ratio between the standard deviation of intervals and the mean interval normalized with respect to a standard mean interval. Calyx units fired rather irregularly (CV^*^ > 0.2) while Bouton units showed a very regular firing behavior (CV^*^ < 0.1). Dimorphic units were modeled with intermediate properties (0.1 ≤ CV^*^ ≤ 0.2). The parameters of the processes which control these regularities in Hayden et al.'s model (i.e., synaptic noise and afterhyperpolarization) were adjusted to chinchillas and thus had to be fitted to human neuron morphology to gain the correct values for CV^*^ in each fiber type. The adapted parameters can be found in Table 3.

**Table 3:** Neuron model parameters for quantal synaptic noise and afterhyperpolarization (AHP) adapted to human vestibular nerve fiber morphology.

		**Fiber type**			**Unit**
		**Calyx**	**Dimorphic**	**Bouton**	
Poisson rate	λ	12	32	1, 250	(0.1ms)^−1^
Quantal amplitude	*g*_*s*_	6.70	4.40	0.28	A m^−2^
AHP amplitude	*g*_*k*_	500	700	1600	A m^−2^
AHP time constant	*g*_*tk*_	2.36	5.00	7.07	ms

*The full set of equations can be found in Hayden (2007) and Hayden et al. (2011)*.

The simulations were conducted using time steps of 0.1 μs. A binary search algorithm scaled the amplitudes of the stimulation protocols systematically to determine the excitation threshold of every nerve fiber. A fiber was elicited if the Na^+^-channel activation parameter *m* rose above 0.7 (Frijns et al., 1994). The termination criterion of the search algorithm was met if upper and lower search boundaries deviated less than 0.1 % from each other.

All ampullary and macular neurons were randomly initialized depending on their fiber types to reflect the stochastic behavior of their respective SDRs. To achieve this, the nerve responses of all three fiber types were simulated and recorded for 10 seconds without external stimulation. Preceding to the onset of stimulus current the initial state of each nerve fiber was chosen as a random point in time out of the appropriate, previously recorded set. Every simulation was conducted 10 times, each with a newly randomized initialization. The averaged recruitment curves were used for further evaluations.

### 2.4. Electrode configurations

All electrode configurations applied in simulations are either monopolar (one source electrode, model boundary as reference) or bipolar (one source and one sink electrode, insulated model boundary).

**Figure 4:**
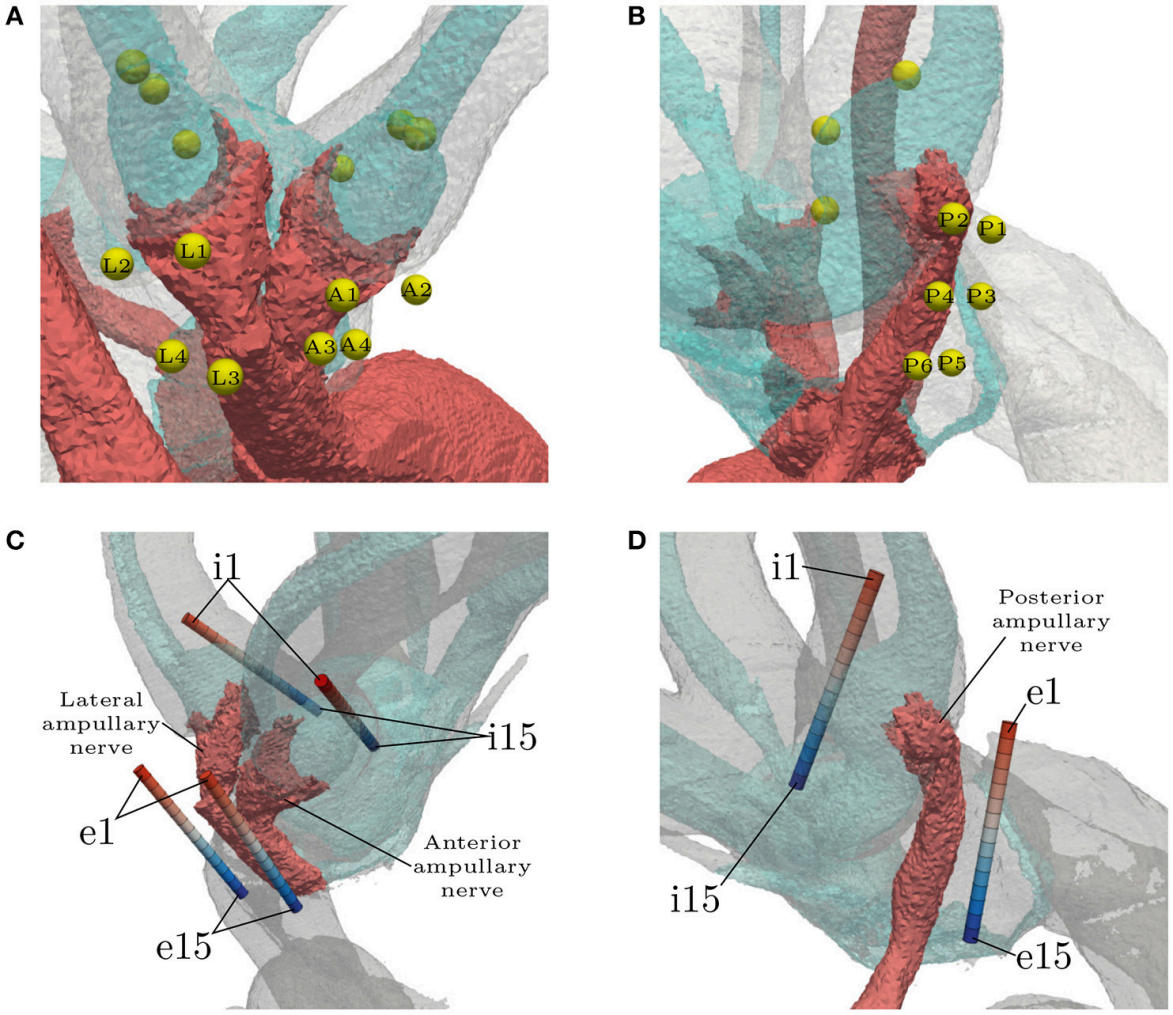
Locations of all spherical **(A,B)** and cylindrical **(C,D)** electrode configurations. The light blue structure shows the endolymphatic duct. EL, spherical configuration labels are abbreviated with respect to their target ampullary nerve (**A**nterior, **L**ateral, **P**osterior). IL, spherical electrodes are unlabeled. EL and IL, cylindrical electrodes (denoted by the prefixes **e** and **i**, respectively) are numbered in ascending order with respect to the insertion depth. The topmost electrodes are labeled with **e1**/**i1** and the deepest with **e15**/**i15**. Optimal placement was assumed to be around the center of the arrays (**e8**/**i8**).

Three IL, spherical electrodes were embedded in every ampulla, each located on the respective endolymphatic duct (see Figures 4A,B). They were not inserted into the endolymphatic duct in order to avoid unnecessary damage to the membranous labyrinth, since the least traumatic implantation process was sought. The first sphere in each ampulla was positioned on top of the respective cupula, roughly along the extension of the central nerve axis where the ampullary nerve connected to the SCC. The second electrode was placed further inside the canal and the third, on the opposite side, in direction of the vestibule. Both of them were also placed on the endolymphatic duct and with a center-to-center distance of 750 μm to the central sphere. Four EL, spherical electrodes were positioned along the anterior and the lateral ampullary nerve, respectively (see Figure 4A). Two spheres of both electrode sets were placed proximal to the sensory epithelia (**A1**, **A2**, **L1**, **L2**) and the rest close to the junction of the two nerves (**A3**, **A4**, **L3**, **L4**). Six electrodes were positioned along the posterior ampullary nerve (see Figure 4B). Starting proximal to the sensory epithelium, they were placed pairwise along the nerve with a center-to-center distance of 750 μm to each other. The distance between each EL, spherical electrode and the nerve surface was 150 μm.

Cylindrical electrode arrays were intended to resemble the estimated insertion path of actual electrodes in clinical practice (see Figures 4C,D). The electrodes with the least insertion depth are labeled with **e1**/**i1**. The insertion depth increases with ascending label numbers up to the deepest inserted electrodes at **e15**/**i15**. They were used to simulate the effects of under- and over-insertion of electrodes in IL and EL locations. Monopolar and bipolar approaches were tested. While using monopolar configurations, every contact in each array was activated solitarily as current source with the model boundary acting as the reference. Bipolar electrode pairs were simulated with a distance of three electrode lengths (0.6 mm) between source and sink. With the current source always in the upper position (lower label number) and the sink in the deeper position (higher label number), the 11 possible bipolar configurations were simulated in each of the 6 arrays, respectively. The arrays were placed with their centers being in the assumed optimal positions for selective stimulation (shortest electrode-nerve distances). IL arrays were arranged, similar to the spherical electrodes, with their centers on top of the cupulas and oriented parallel to the endolymphatic ducts, while not penetrating them. EL insertion paths were chosen along the respective nerves with the lower half of the array approximately parallel to the central axes of the nerves.

Because of the less traumatic positioning outside of the endolymphatic duct, all electrodes placed in the IL approaches were located relatively far from their respective target nerves. To test the effects of a closer stimulation site, three additional spherical electrodes were inserted into the cupula; one centrally above each sensory epithelium with a distance of 150 μm, respectively.

### 2.5. Stimulation waveforms

**Figure 5:**
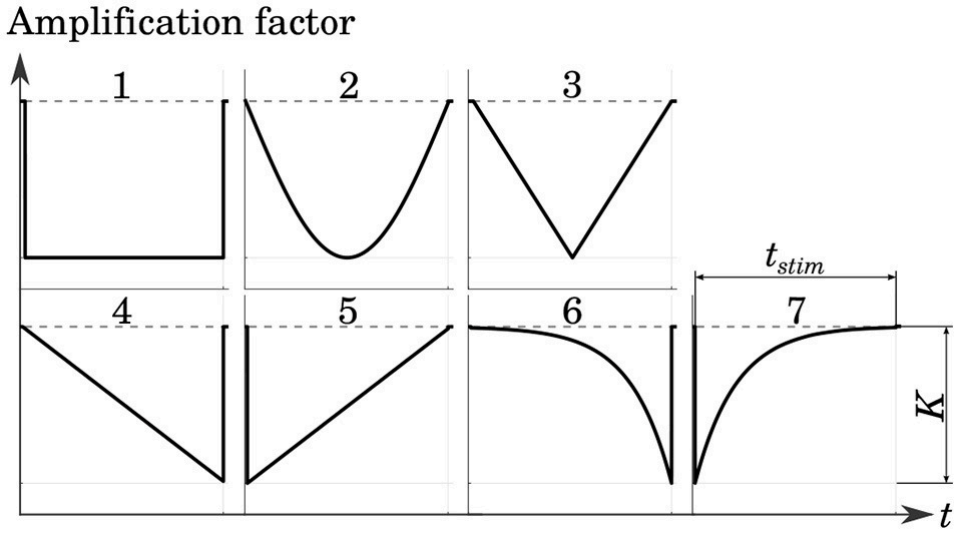
Tested cathodic stimulation waveform shapes: rectangular (1), sinusoidal (2), centered triangular (3), linearly increasing (4) and decreasing (5), exponentially increasing (6) and decreasing (7). All but the centered triangular waveform were adopted from Sahin and Tie (2007). Stimulus phase duration *t*_*stim*_ and amplitude *K* were varied in the simulations.

Several cathodic waveform shapes with a number of different pulse durations were tested in order to determine a stimulation protocol which yielded good selectivity while maintaining low levels of energy consumption. The shapes of the stimulation phases were adopted from Sahin and Tie (2007). This included rectangular, sinusoidal as well as linearly and exponentially increasing and decreasing pulses. The Gaussian waveform was approximated with a centered triangular pulse. All pulse shapes are shown in Figure 5.

During neural stimulation it is necessary to restore the electrochemical balance of the tissue after each pulse to avoid damage (Mortimer et al., 1970; Robblee and Rose, 1990; Merrill et al., 2005). This was done with a charge-recovery phase of opposite polarity following the stimulation pulse. Pseudomonophasic pulses (short and strong stimulation phase, followed by a long charge-recovery phase with smaller amplitude) were used for all stimulations since they provide better selectivity results than symmetric biphasic pulses (Hayden et al., 2011). All stimuli were charge-balanced using rectangular recovery phases of opposite polarity with their amplitude being 20 % of the stimulation phase peak amplitude *K*. The stimulation phase duration *t*_*stim*_ was varied in a range between 10 and 500 μs. From 10 to 100 μs *t*_*stim*_ was incremented by 10 μs steps, from 100 to 200 μs in 20 μs steps and from 200 to 500 μs in 50 μs steps. The recovery phase duration was chosen accordingly to achieve a balanced charge injection.

Energy consumption was evaluated based on the stimulation phase of the stimulus protocol required to activate 80 % of target nerve fibers. The root mean squares of both current *I*_*RMS*_ and voltage *V*_*RMS*_ between source electrode and ground were calculated from the accordingly scaled stimulation phase. Required energy *E* was computed by the equation *E* = *I*_*RMS*_ · *V*_*RMS*_ · *t*_*stim*_.

### 2.6. Selectivity evaluation

**Figure 6:**
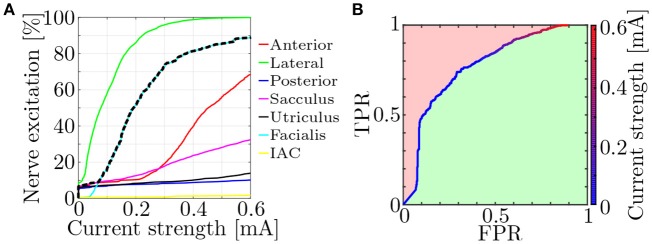
**(A)** Exemplary fiber recruitment curves (Hayden et al., 2011) resulting from a monopolar, EL electrode near the lateral ampullary nerve and **(B)** the corresponding ROC curve. Based on the dashed, black line in **(A)**, which depicts the highest percentage of excited non-target nerves, the FPR is computed. The green space in **(B)** represents the AUC and is used as a measure for the worst-case selectivity of the electrode configuration.

Fiber recruitment and nerve selectivity depend on a variety of parameters (e.g., nerve morphology, electrode configuration, stimulus shape). Approaches to quantify the selectivity of neural stimulation were already published (Schiefer et al., 2010; Raspopovic et al., 2011). They compared the target nerve recruitment to the mean of the entire non-targeted recruitment. However, since a single activated non-target nerve is also potentially disruptive to efficient restoration of vestibular function, a measure to objectively evaluate the *worst-case selectivity* using the fiber recruitment curves (see Figure 6A) was introduced.

Receiver operating characteristic (ROC) curves were produced by plotting the true positive rate (TPR) of the nerve excitation against the false positive rate (FPR) during a stepwise increase of the applied stimulation current (see Figure 6B). The TPR was calculated in every step as the ratio between the number of activated target nerve fibers and all fibers in the target nerve. FPRs were computed analogously for each non-target nerve but only the highest FPR value in every current step was used for plotting the ROC curves to evaluate the selectivity of the worst-case scenario (dashed, black line in Figure 6A).

The selectivity of a stimulation was determined by numerical integration of the ROC curve. This yielded the size of the area under the curve (AUC) and lay between 0 and 1. While 0 implied no activation of target nerve fibers at all and 1 the optimal selectivity, an AUC above 0.5 was always desired, since lower values indicated a stronger excitation of non-target nerves than target nerves. It is generally possible to swap positive and negative binary classifiers, correcting the AUC values to always lie above 0.5. However, since a swapping of these parameters would also alter the meaning of the ROC curves in this case (activated neurons becoming non-activated neurons and vice versa), no correction was applied. Each simulation was conducted with the goal to maximize AUC.

All simulations analyzing the selectivities of different electrode configurations were performed using an anodic and a cathodic, pseudomonophasic protocol. Stimulation phases were always rectangular pulses lasting 100 μs.

## 3. Results

### 3.1. Electrode configuration dependency

#### 3.1.1. Spherical electrodes

Most EL, monopolar electrodes showed high selectivities. While an AUC value of 0.64 was achievable in the anterior nerve, the lateral and posterior nerves exhibited values of 0.85 and 0.88, respectively (see Table 4). Electrodes more distant to the ampulla produced better selectivities than closer ones. Cathodic stimulation resulted in slightly higher maximum AUC values and in average 52.2 ± 7.0% less energy expenditure in all spherical, monopolar simulations. During stimulations of the lateral and the posterior nerve, the simulated recruitment curves showed that almost 30 % of the target nerve fibers were selectively stimulated by low currents without excitation of non-targeted nerves. In contrast, excitation of targeted and non-targeted nerves could not be separated at all with application of IL, monopolar stimulation. The saccular and utricular nerves got stimulated in the same current ranges as the target nerves in every IL, monopolar stimulation approach.

**Table 4:** AUC values of EL, spherical electrode stimulation with anodic and cathodic, pseudomonophasic pulses and the stimulus energy *E*_80_ required for 80 % target nerve activation.

**Electrode no**.	**Cathodic AUC /** ***E***_**80**_**[μJ]**			**Anodic AUC /** ***E***_**80**_**[μJ]**		
	**Anterior**	**Lateral**	**Posterior**	**Anterior**	**Lateral**	**Posterior**
1	0.526/0.316	0.811/0.187	0.845/0.156	0.431/0.824	0.778/0.507	0.825/0.415
2	0.466/1.175	0.292/0.978	0.642/0.746	0.367/2.260	0.317/1.633	0.633/1.434
3	0.503/0.138	**0.848/0.064**	0.875/0.086	0.471/0.252	0.795/0.151	0.870/0.169
4	**0.640/0.319**	0.755/0.086	0.856/0.132	0.591/0.722	0.807/0.188	0.849/0.284
5	–	–	**0.878/0.064**	–	–	0.875/0.116
6	–	–	0.877/0.060	–	–	0.875/0.113

*Bold numbers indicate the best selectivity achieved in the respective nerve. The electrode numbers refer to the labels depicted in Figures 4A,B*.

Spherical, monopolar electrode configurations at IL locations yielded unsatisfactory selectivity results. No AUC exceeded a value of 0.54, which was achieved in the posterior SCC by the electrode located furthest inside the canal. Stimulations in anterior and lateral SCCs were both unable to reach AUC values of 0.5. In all cases the saccular nerve exhibited the strongest non-targeted stimulation, followed by the utricular nerve.

During stimulation with the three additional electrodes placed close to the sensory epithelia the AUC values increased to 0.55, 0.56, and 0.59 for the anterior, the lateral and the posterior ampullary nerve, respectively. However, whether this moderate improvement of performance justifies damaging the membranous labyrinth or not, needs to be weighed thoroughly.

#### 3.1.2. Cylindrical electrode arrays

**Figure 7:**
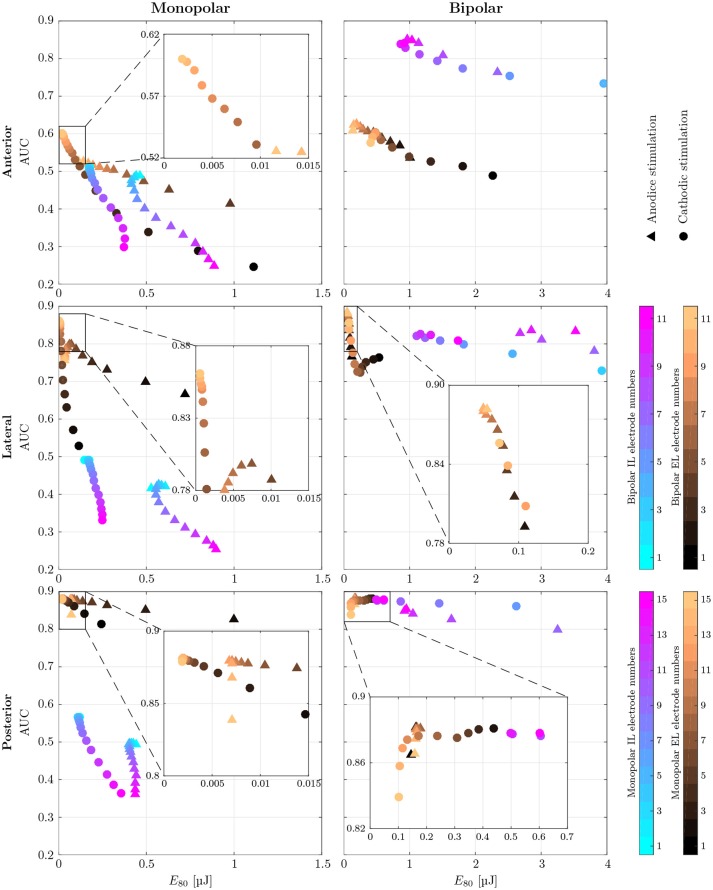
AUC values of monopolar **(Left)** and bipolar **(Right)** stimulation with IL and EL, cylindrical electrodes (color-coded) using anodic (triangles) and cathodic (circles), pseudomonophasic pulses plotted against the stimulus energy *E*_80_ required for 80 % target nerve activation. Targeted were the anterior (top row), the lateral (central row) and the posterior (bottom row) ampullary nerves. The color-coded electrode numbers refer to the labels depicted in Figures 4C,D. Bipolar electrode numbers refer to the current source. The current sink was chosen as described in section 2.4. Regions of interest with high AUC values and low energy consumption are presented in a detailed view. EL electrode configurations show reduced energy expenditure compared to IL configurations throughout all simulations. Also, higher AUC values are achieved by EL electrodes in all but the anterior bipolar stimulation approach.

**Figure 8:**
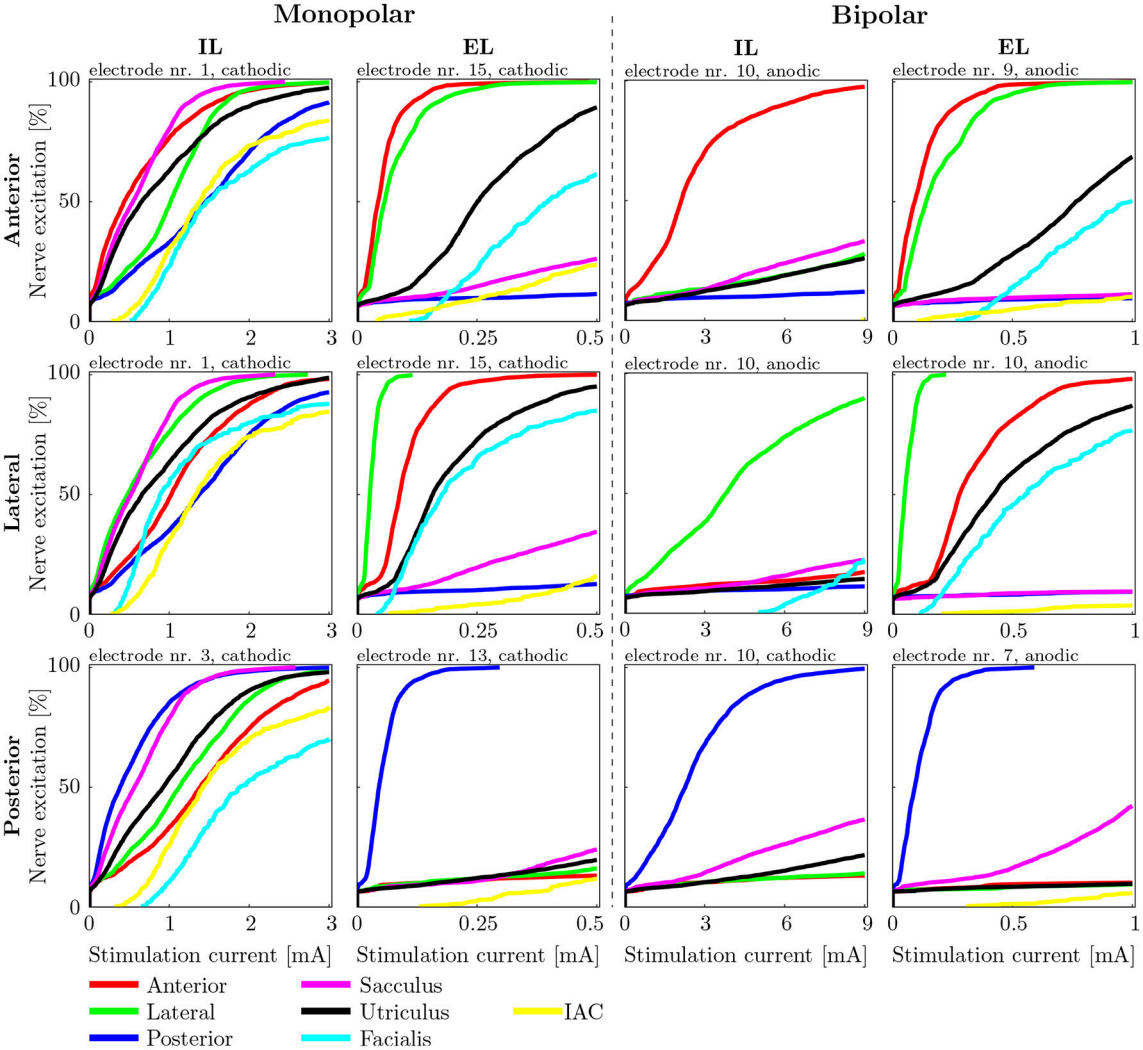
Recruitment curves of the electrodes with the highest AUC values during stimulation of the anterior **(Top)**, the lateral **(Central)**, and the posterior **(Bottom)** ampullary nerves. The four columns depict the different IL and EL, monopolar and bipolar electrode configurations. The graphs are labeled on top with the electrode configuration and stimulation polarity which produced the respective recruitment curves.

AUC values as well as the stimulus energy *E*_80_ required for 80 % target nerve activation from all configurations of the mono- and bipolar, IL and EL, cylindrical electrodes are presented in Figure 7. The corresponding recruitment curves with the highest AUC values of each electrode configuration (mono- and bipolar, IL and EL) in all three target nerves are depicted in Figure 8 for visual inspection of the stimulation.

Monopolar stimulation yielded better results with cathodic instead of anodic pulses in all cases. The evaluation of AUCs in the IL, monopolar approaches predicted a deterioration of selectivity with deeper insertion into the vestibule. While there were only minimal changes in selectivity when the electrode was moved within the canal (Figures 4C,D, **i1** to **i6**), the decrease of AUC when inserting the electrode further into the vestibule (**i7** to **i15**) was much stronger. The maximum selectivities of IL, monopolar approaches were found near the top of each array (**i1** to **i3**) with cathodic stimulation pulses.

When using EL, monopolar configurations, deeper electrodes (**e9** to **e15**) showed higher AUC values. Conversely to the IL, monopolar approach, the electrode positioning was rather uncritical in the lower part of the array (**e9** to **e15**). No large variations of selectivity were observed when stimulating the anterior and lateral ampullary nerves with these parts of the arrays. The upper halves of these arrays showed strongly decreased selectivity. In the posterior ampullary nerve AUC values were similar throughout the entire array while varying the insertion depth of the EL, monopolar electrodes. EL, monopolar electrodes yielded highly selective stimulation results in the lateral and posterior ampullary nerves. However, AUC values did not exceed 0.6 when stimulating the anterior nerve branch. Nonetheless, EL, cathodic stimulation outperformed all other monopolar configurations in terms of selectivity as well as energy expenditure.

**Figure 9:**
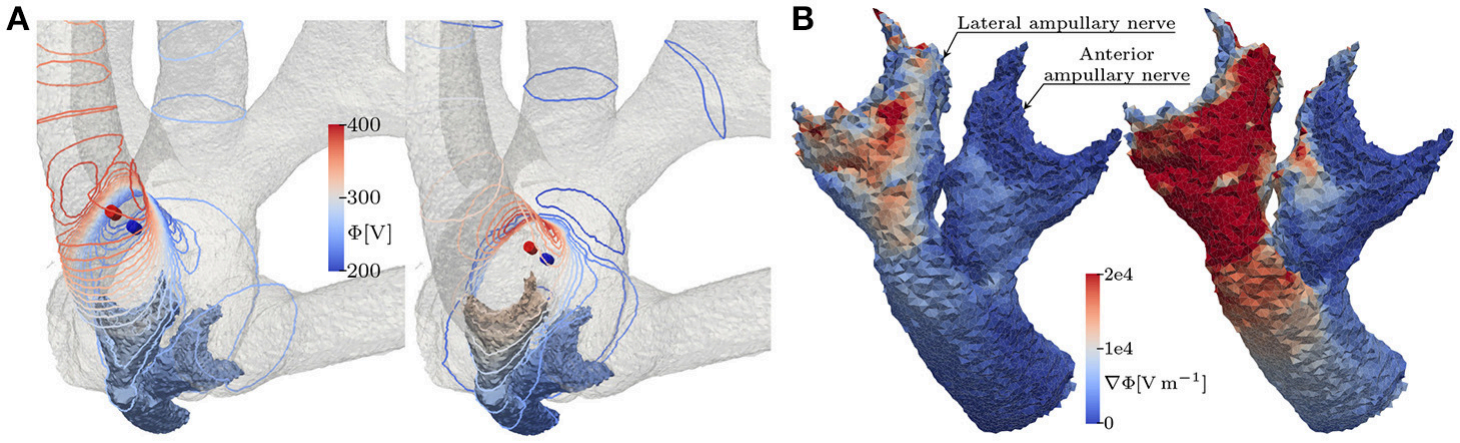
**(A)** Equipotential lines at the surface of the vestibular system and **(B)** the resulting potential gradient along anterior and lateral nerve of the topmost (left) and the deepest (right) lateral, bipolar electrode configuration, respectively. The fields are derived from the unscaled potential distribution with a current strength of 1 A from active to reference electrode (from red to blue cylinders).

Bipolar, IL electrode configurations showed much higher AUC values than the analogous monopolar approaches. An inversion of the insertion depth dependency compared to the monopolar equivalent was observed here. Deeper inserted bipolar electrodes exhibited superior selectivities (0.81 < AUC < 0.88) as opposed to configurations located on top of the arrays (0.53 < AUC < 0.63). The deeper inserted electrodes produced larger potential gradients along the target nerves (see Figure 9). It was possible to achieve very selective stimulation results in all three ampullary nerves by the use of IL, bipolar electrodes.

No considerable improvement of maximum selectivity was observable by the use of bipolar instead of monopolar electrodes in the EL space. Insertion depth dependency was analogous to the monopolar approach. Interestingly, most EL, bipolar stimulations yielded slightly better AUC values and reduced energy consumptions with application of anodic instead of cathodic pulses.

Energy consumption *E*_80_ varied strongly over the different electrode arrangements and locations. For every configuration, the electrode requiring the least amount of energy was determined in each target nerve, respectively. On average, monopolar contacts consumed 84.4±4.0% less energy than analogous bipolar electrodes. Also, EL stimulation showed 89.1 ± 5.6% reduced energy expenditure compared to related IL approaches. Monopolar, EL electrodes were the most energy efficient, requiring cathodic stimulation pulses around a mean of *E*_80_ = 14.4nJ. Monopolar, IL and bipolar, EL electrodes showed energy consumptions with averages of *E*_80_ = 143.1nJ and *E*_80_ = 97.4nJ, respectively. The highest amounts of energy were required by bipolar, IL configurations with a mean of *E*_80_ = 826.2nJ per pulse.

### 3.2. Variation of electrical stimulation patterns

**Figure 10:**
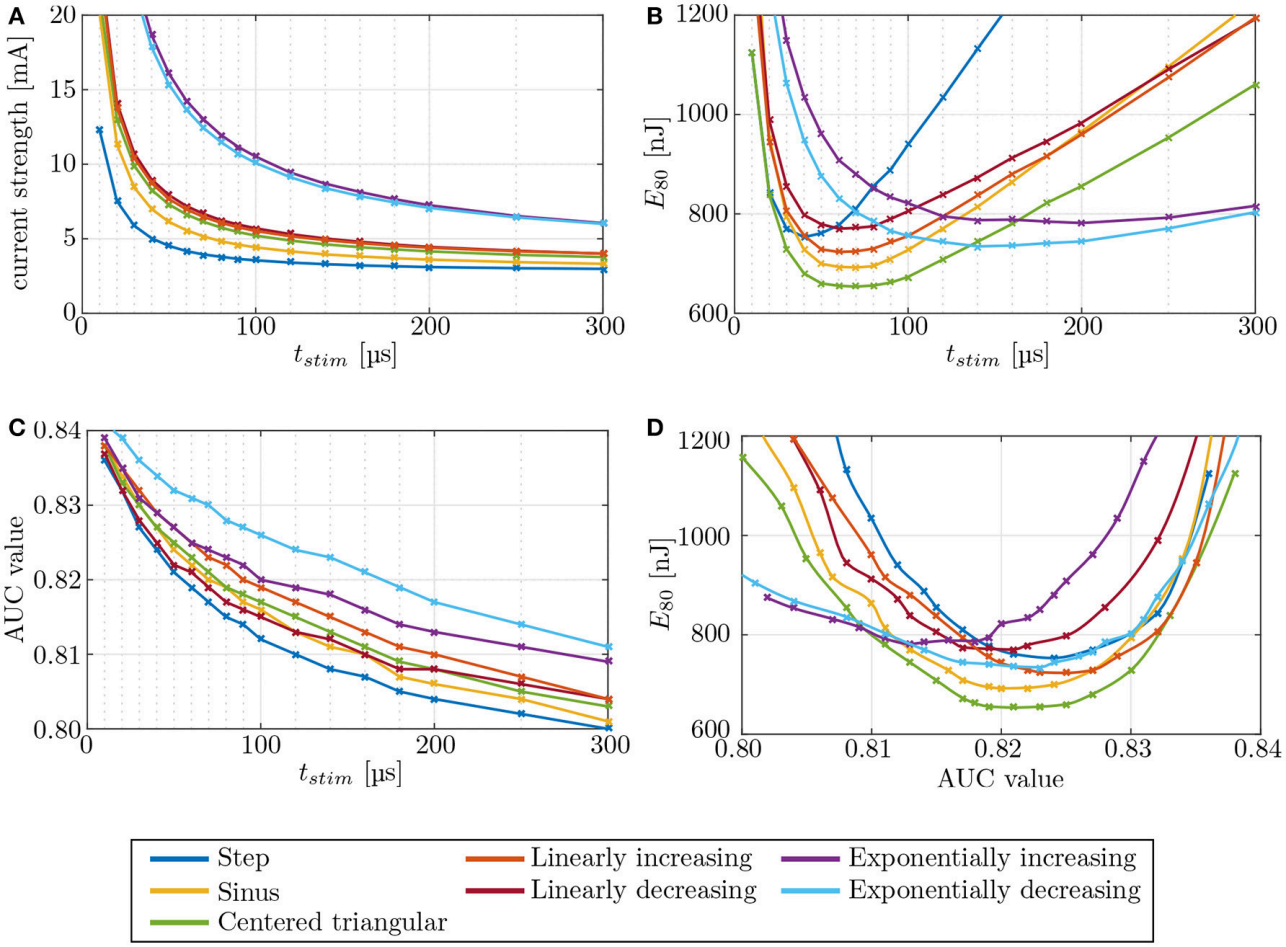
Evaluation of different stimulation waveforms of the electrode configuration **i11**–**i15**, located in the anterior SCC. Stimulation phase duration is systematically increased from 10 μs to 500 μs. Since phase durations longer than 300 μs did not offer any advantages, they are not displayed. Phase duration dependencies of **(A)** current strength, **(B)** energy, and **(C)** selectivity were recorded. The strength-duration curve and the consumed energy *E*_80_ were computed at (80 %) target nerve activation. Panel **(D)** shows the required energy expenditure to reach a certain level of selectivity. The crosses mark the different pulse durations.

Stimulation pulse waveforms were varied in mono- and bipolar, IL and EL electrode configurations to determine their effect on selectivity and energy consumption. Required current strength, expended energy and AUC values were recorded with respect to the stimulation pulse durations for all waveforms (see Figure 10). Also, energy was plotted against the AUC value to determine waveforms, which reached high selectivities while maintaining low energy levels. Different electrode configurations yielded qualitatively very similar results when compared among each other.

The strength-duration curves in Figure 10A were recorded for 80 % stimulation of the target nerve fibers, using an IL, bipolar electrode (**i11** - **i15**) in the anterior SCC. The step pulse waveforms required the least current strengths for the stimulation while exponential pulses showed the highest amplitudes. All other pulse shapes injected slightly higher currents than the step pulse. However, our simulations identified the centered triangular pulse as the most energy-efficient waveform up to at least *t* = 136 μs in all simulations (see Figure 10B). Exponentially increasing as well as decreasing waveform shapes were more efficient when using longer pulses. The minimum energy expenditure of each waveform shape was observed at their respective chronaxie times.

Variations of the pulse shape offered no considerable advantages regarding the achieved selectivity (see Figure 10C). At their respective chronaxie pulse durations, the AUC values of the individual waveforms did not deviate more than ±3 % from each other. However, it was apparent throughout all waveform shapes, that shorter stimulation times yield slightly higher AUC values than longer pulses. The step pulse reached one of the highest selectivities of all waveform shapes at chronaxie pulse duration but all other shapes, except for linearly decreasing and exponentially increasing pulses, performed better than the step pulse when not constrained to the minimum energy achievable by each waveform, respectively (see Figure 10D). Higher AUC values at lower amounts of required energy were achieved. The centered triangular pulse outperformed every other waveform shape by consuming the least energy to reach the same extent of selectivity up to pulse durations around 140 μs. Exponentially increasing and decreasing waveform shapes were favorable when using long stimulation pulse durations, but since shorter pulses were superior in terms of selectivity and energy consumption, exponential pulse shapes were not reasonable in this scenario. The centered triangular pulses expended in average 13.2 % less energy than step pulses at their respective chronaxie pulse durations.

## 4. Discussion

### 4.1. Model predictions

Our goal was to find electrode configurations and stimulation sites in which the selectivity is as prominent as possible to ensure undistorted stimulation results. By calculating the AUC value we quantify this very characteristic. Therefore, the higher the AUC values are the higher are the chances of success for a potential restoration of normal vestibular function. We believe that this is a good indicator for the restoration quality. Vast differences between IL, EL, monopolar and bipolar electrodes in both achievable selectivity and expended energy were identified (see Table 4 and Tables A1,A2 in Supplementary Material as well as Figures 7, 8). Our results were obtained using spherical as well as cylindrical electrodes. Due to their geometry there were relevant differences of volume and surface area between the two electrode shapes. However, since the nerve stimulations were exclusively compared among identical electrode shapes, the mismatch in size is irrelevant.

On average IL, bipolar electrodes reached the highest AUC values, but at the cost of immensely increased energy consumption compared to all other configurations. In contrast, IL, monopolar contacts showed by far the worst performance regarding selectivity and also required more stimulation energy than both EL approaches. The highly conductive peri- and endolymphatic fluids produced a wide current propagation throughout the vestibular system which rendered selective stimulation almost impossible. Our results regarding EL, bipolar electrode configurations indicated that they were an unfavorable choice. No improvement of selectivity, but a gain of energy expenditure compared to the EL, monopolar approach was observed. The use of EL, monopolar electrodes yielded advantages in every aspect compared to IL, monopolar configurations. Both improved selectivity and reduced energy consumption were visible. Because of the low selectivity of IL, monopolar electrodes and the highly increased energy expenditure of bipolar configurations, we conclude that the EL, monopolar option is the most promising approach for a functional vestibular implant according to our simulations.

Conversely to the results of Marianelli et al. (2015), better selectivities of EL electrodes were achieved when stimulating with deeper contacts, further away from the ampulla. Especially in the anterior and lateral ampullary nerves the achievable selectivity was sensitive to misplacement in the upper halves of the electrode arrays (**e1** to **e8**). The curvatures of these nerves caused greater distances between the upper electrodes and the respective target nerves (see Figure 4C) and thus selective stimulation was barely possible with those contacts. The posterior ampullary nerve was almost straight (see Figure 4D), which resulted in an invariable electrode-nerve distance and a rather uncritical insertion depth. However, also spherical electrodes, which were placed with a fixed distance between the contacts and the nerve surfaces, yielded higher selectivities when located further away from the ampulla. EL electrodes near the ampulla are also close to the liquid filled bony labyrinth, which encourages a stronger current propagation throughout the vestibular system due to its high conductivity. Electrodes more distant from the ampulla are surrounded mostly by low conductive bone, which causes a more concentrated current flow through the target nerve.

Other than the monopolar approach, IL, bipolar contacts offered better selectivities with deeper inserted contacts. This was due to the fact, that deeper inserted bipolar electrodes produced larger variations of the potential gradient along the target nerves (see Figure 9). Nerve fibers are easily excitable by those variations, which is also evident in the neuron model equations as larger extracellular potential gradients along the nodes of Ranvier produce stronger internodal currents (Frijns et al., 1994). Since current flow of bipolar electrodes is quite directional, positioning and especially orientation is more critical compared to monopolar contacts. This dependency on the potential gradient was also the reason why EL stimulation yielded lower AUC values in the anterior ampullary nerve than in the other two branches. The anterior nerve branch was thicker than the neighboring lateral branch in our dataset. Due to the steeper potential gradient along thinner volumes the lateral ampullary nerve was more sensitive to the EL stimulation than the anterior branch.

A direct estimation about critical stimulation scenarios with respect to facial nerve excitation can not be given since it is not fully clear at which percentage of stimulated facial neurons the nerve is innervated. However, the results of Guinand et al. (2015) report upper comfortable levels at which the facial nerve is being stimulated between 200 μA and >550 μA during IL and EL monopolar stimulation, respectively. This suggests that, according to the simulated fiber recruitment curves in Figure 8, a rather low percentage of innervated facial neurons (around 10 %) can already lead to undesired facial twitching in the worst case.

The distance between current source and current sink in bipolar configurations plays a crucial role with respect to their energy expenditure. More space between the two electrodes results in a less directional current flow from source to sink. This considerably reduces the required current strength to excite the target nerve. In this regard, an optimal electrode distance for a good trade-off between energy consumption and selectivity has yet to be found.

The different stimulation pulse waveforms showed no major variations regarding selectivity at their respective chronaxie times (see Figures 10A,C). However, the achieved AUC values increased progressively by the use of shorter stimulus durations. This effect was also reported by Davidovics et al. (2011). As published by Sahin and Tie (2007) and Foutz and McIntyre (2010), the least energy expenditure can be expected from sinusoidal, Gaussian as well as centered triangular pulses in a wide range of short pulse durations. Our simulations confirmed these results and identified the centered triangular pulse as the most energy-efficient of the investigated waveform shapes. When comparing the achieved selectivity at equal amounts of expended energy among the different waveform shapes, the centered triangular pulse was superior to all other pulse shapes up to 136 μs in every simulation. A genetic algorithm by Wongsarnpigoon and Grill (2010) showed that truncated Gaussian pulses could even outperform all of the waveform shapes used in this study. However, the rather complex pulse shape would probably require a more sophisticated and energy-consuming circuit design, which could nullify the predicted improvement.

### 4.2. Model uncertainties

Our model was validated by simulating stimulation scenarios similar to Hayden et al.'s *virtual labyrinth* model (Hayden, 2007; Hayden et al., 2011). They were able to predict eye rotations caused by electric stimulation of vestibular nerves in chinchilla. Since Hayden et al.'s model was fitted to chinchilla anatomy, considerable developments were conducted in order to adapt the model toward human anatomy. In this respect, the different fiber morphology of human neurons causes a disturbance in the firing regularity. Regarding the SDR, the firing regularities of the different fiber types were adapted to the range of the normalized coefficient of variation as described by Goldberg et al. (1984). These adaptations are required to determine a correct initial state for the neuron excitation. The obtained “starting points” for the excitation are solely dictated by the nature of the SDR and the adapted normalized coefficient of variation. Thus, the adaptations do not effect the underlying neuron model. Therefore, we consider the validation of the model referring to the literature justified although considerable differences are introduced into the model. Qualitatively, our simulations showed a high resemblance to the results from literature. However, quantitatively, a notable difference was observed (Handler et al., 2017). This was expected, since there was a large mismatch of size, fiber morphology and the surrounding of the vestibular system between the compared anatomical datasets (i.e., stimulations in a human inner ear were compared to results obtained by simulations based on anatomical models of chinchillas; Hayden et al., 2011). To date, no experimental data to appropriately validate the model is available.

The FEM model had to be simplified in several aspects to minimize the computational load. Surrounding components of the vestibular system, such as brain, middle ear, and mastoid cells, were all approximated with the enclosing bone- and saline spheres. For bipolar electrode configurations, this simplification should not have had a great effect on the outcome of the simulations. Current source and sink were close to each other, which ensured a rather concentrated potential drop. However, since the entire surface of the saline sphere was modeled as a current sink (i.e., the reference electrode) when simulating monopolar electrodes, the simplified surroundings could have altered the natural current propagation considerably.

The entire stimulation range from 0 to 100 % percent target nerve activation has been taken into account in the calculation of the AUC values during all simulations. This might not be entirely reasonable in practice since a fully activated target nerve would correspond to extreme head movements which is eventually not necessary for re-establishment of natural vestibular function. However, there is no experimental data or references—to our knowledge—giving suggestions for a default stimulation rate or threshold in the ampullary nerves. Although publications concerning the stimulation via prototypical implants yield some current amplitudes and/or current ranges in which eye movements have been observed (Guinand et al., 2015), these current ranges vary strongly across the experiments since anatomy, implantation site and the stimulation device are not uniform. Thus, no reasonable estimate for a meaningful excitation threshold could be computed and the entire stimulation range of the target nerve was used for selectivity comparisons.

Another aspect about the AUC is that it neglects some important information of the data for the sake of comparability of the recruitment curves. The main motivation for using the AUC was the feasibility of quantification of the selectivity which was necessary to objectively compare the stimulation results. However, the AUC does not take into account which non-target nerve is used for the computation of the FPR, leading to the incorrect assumption that all undesired excitation is equally problematic. This is not the case because, in general, a stimulation of the facial nerve would be worse than the elicitation of a non-targeted ampullary nerve. Nonetheless, a weighting for stimulation of different non-target nerves was not introduced since the determination of appropriate weights was not possible due to missing data on this subject. A second problem is that two entirely different sets of recruitment curves may produce the same AUC values. Therefore, the AUC value is only expected to serve as a guidance parameter and the underlying recruitment curves always have to be examined when choosing electrode configuration and stimulation site.

The potential distributions were computed using purely resistive components, neglecting the time- and frequency-dependency of the tissue conductivity. A study about tissue impedance and current flow in the cochlea showed, that the assumption of quasi-static conditions holds for frequencies up to 12.5 kHz (Spelman et al., 1981). Thus, due to the similar environment in the vestibular system, the entire potential distribution was assumed to be linearly scalable using arbitrary stimulation pulses as long as the majority of their spectral energy lay below this threshold. Whereas this assumption was well-justified for stimulus pulse durations *t*_*stim*_ > 50 μs, a major part of the spectral energy lay above this frequency-threshold when applying shorter pulses (Hayden et al., 2011). Therefore, the results for short pulses are not as reliable as simulations of longer pulse durations. Additionally, the electrode-tissue interface impedance was not considered in the computation of energy expenditure. This could also cause a quantitative deviation between simulated and *in vivo* stimulation.

Implementing a refined surrounding of the vestibular system and defining a more realistic reference for monopolar stimulation (e.g., adding a reference electrode behind the auricle) are the next steps to improve the credibility of the simulations. Also, a more sophisticated model validation and simulation results on the basis of more than one dataset are necessary. With the aid of a realistic surrounding, appropriate boundary conditions and a credible *in vivo* validation method, we hope to establish a highly accurate simulation framework to support the development of a safe and efficacious vestibular implant.

## Ethics statement

This study was carried out in accordance with the recommendations according to the guidelines of the ethical review committee of the Medical University of Innsbruck with written informed consent from all subjects. All subjects gave written informed consent in accordance with the Declaration of Helsinki. The protocol was approved by the ethical review committee of the Medical University of Innsbruck.

## Author contributions

PS, implementation of electrical model, experiment design, and evaluation of results. MH, meshing, implementation FEM, experiment design, and evaluation of results. LJ, specimen processing, contrast enhancement, manual segmentation of 33 temporal bones, and data interpretation. AS-F, method development for contrast enhancement and imaging, specimen collection over 10 years, specimen preparation, setting of anatomical landmarks, and validation of segmentations. KF, analysis, processing, and interpretation of image data. RS, experiment design, organization. CB, experiment design, organization, and scientific consulting. DB, experiment design, interpretation of data, organization, and scientific consulting. All authors were responsible for drafting and/or revising the manuscript. Additionally, they approved the final version of this document and agreed upon the accountability for all aspects of this work.

### Conflict of interest statement

RS works as a research engineer for MED-EL GmbH in Innsbruck, Austria.

The remaining authors declare that the research was conducted in the absence of any commercial or financial relationships that could be construed as a potential conflict of interest.
