# Supplementary material for: Model-Based Vestibular Afferent Stimulation: Evaluating Selective Electrode Locations and Stimulation Waveform Shapes
Source: Front Neurosci. 2018 Aug 30;12:588. doi: 10.3389/fnins.2018.00588 (PMC6125370; doi:10.3389/fnins.2018.00588)
Supplement: Supplementary file 1 [file Data_Sheet_1.pdf]

Table A1. AUC values of monopolar, IL and EL, cylindrical electrode stimulation with anodic and cathodic, pseudomonophasic pulses and the stimulus energy E_{80} required for $80\,\%$ target nerve activation. This table is also presented in graph form in Figure 7. Bold numbers indicate the best selectivity achieved in the respective nerve and electrode configuration. The recruitment curves of these configurations is depicted in Figure 8. The electrode numbers refer to the labels depicted in Figures 4c and 4d.

Monopolar electrodes						
Electrode no.	Cathodic AUC / E_{80}[µJ]			Anodic AUC / E_{80}[µJ]		
	Anterior	Lateral	Posterior	Anterior	Lateral	Posterior
**i1**	**0.512/0.182**	**0.490/0.148**	0.565/0.121	0.488/0.462	0.417/0.526	0.493/0.441
**i2**	0.512/0.176	0.491/0.172	0.566/0.117	0.488/0.441	0.422/0.606	0.495/0.426
**i3**	0.510/0.174	0.490/0.169	**0.567/0.113**	0.484/0.429	0.425/0.584	0.496/0.413
**i4**	0.506/0.175	0.488/0.170	0.566/0.112	0.477/0.418	0.425/0.570	0.496/0.406
**i5**	0.501/0.177	0.483/0.172	0.563/0.113	0.465/0.416	0.422/0.561	0.494/0.401
**i6**	0.493/0.182	0.476/0.177	0.558/0.115	0.448/0.430	0.413/0.555	0.489/0.403
**i7**	0.482/0.193	0.466/0.184	0.551/0.120	0.425/0.450	0.398/0.557	0.482/0.404
**i8**	0.468/0.206	0.453/0.194	0.539/0.126	0.401/0.492	0.378/0.572	0.473/0.408
**i9**	0.450/0.220	0.436/0.201	0.524/0.139	0.377/0.552	0.354/0.607	0.460/0.415
**i10**	0.429/0.254	0.417/0.212	0.504/0.159	0.354/0.642	0.332/0.664	0.446/0.426
**i11**	0.403/0.296	0.398/0.227	0.478/0.186	0.332/0.712	0.311/0.719	0.429/0.433
**i12**	0.376/0.338	0.378/0.239	0.446/0.225	0.309/0.778	0.293/0.780	0.410/0.441
**i13**	0.348/0.369	0.360/0.246	0.414/0.275	0.287/0.823	0.277/0.844	0.391/0.437
**i14**	0.321/0.376	0.345/0.248	0.386/0.315	0.266/0.854	0.264/0.883	0.375/0.437
**i15**	0.298/0.374	0.332/0.248	0.364/0.358	0.248/0.886	0.253/0.898	0.361/0.437
**e1**	0.246/1.111	0.528/0.117	0.813/0.244	0.220/8.020	0.665/0.720	0.784/2.075
**e2**	0.289/0.796	0.572/0.083	0.842/0.147	0.260/5.872	0.698/0.495	0.827/0.998
**e3**	0.338/0.512	0.630/0.048	0.861/0.089	0.311/3.356	0.732/0.274	0.851/0.495
**e4**	0.390/0.329	0.666/0.034	0.871/0.056	0.361/1.856	0.751/0.190	0.863/0.276
**e5**	0.448/0.210	0.704/0.025	0.876/0.042	0.413/0.977	0.770/0.138	0.870/0.190
**e6**	0.491/0.152	0.744/0.018	0.878/0.032	0.452/0.627	0.787/0.100	0.874/0.137
**e7**	0.513/0.119	0.781/0.014	0.879/0.026	0.475/0.485	0.798/0.075	0.876/0.105
**e8**	0.531/0.096	0.807/0.012	0.880/0.023	0.491/0.391	0.798/0.060	0.878/0.088
**e9**	0.549/0.076	0.826/0.010	0.880/0.022	0.503/0.314	0.792/0.050	0.878/0.080
**e10**	0.560/0.063	0.841/0.009	0.881/0.021	0.507/0.277	0.786/0.043	0.879/0.074
**e11**	0.568/0.050	0.850/0.009	0.881/0.020	0.510/0.235	0.780/0.038	0.879/0.071
**e12**	0.579/0.039	0.852/0.008	0.881/0.020	0.516/0.194	0.775/0.036	0.879/0.067
**e13**	0.591/0.032	0.854/0.007	**0.881/0.020**	0.522/0.171	0.769/0.034	0.878/0.071
**e14**	0.598/0.024	0.857/0.006	0.881/0.019	0.525/0.143	0.763/0.034	0.868/0.071
**e15**	**0.600/0.019**	**0.861/0.005**	0.879/0.019	0.525/0.117	0.755/0.036	0.839/0.071

Table A2. AUC values of bipolar, IL and EL, cylindrical electrode stimulation with anodic and cathodic, pseudomonophasic pulses and the stimulus energy E_{80} required for $80\,\%$ target nerve activation. This table is also presented in graph form in Figure 7. Bold numbers indicate the best selectivity achieved in the respective nerve and electrode configuration. The recruitment curves of these configurations is depicted in Figure 8. The first electrode numbers indicate the current sources and the second the current sinks. The electrode numbers refer to the labels depicted in Figures 4c and 4d.

Bipolar electrodes							
Electrode no.		Cathodic AUC / E_{80}[µJ]			Anodic AUC / E_{80}[µJ]		
		Anterior	Lateral	Posterior	Anterior	Lateral	Posterior
**i1 -**	**i5**	0.626/38.031	0.525/13.909	0.477/102.899	0.545/20.775	0.542/25.410	0.658/25.217
**i2 -**	**i6**	0.676/14.551	0.644/6.683	0.588/44.637	0.591/14.749	0.593/17.294	0.666/20.756
**i3 -**	**i7**	0.712/6.760	0.728/3.924	0.758/13.214	0.623/10.227	0.622/13.733	0.695/15.667
**i4 -**	**i8**	0.734/3.952	0.775/2.570	0.830/5.255	0.648/7.711	0.657/10.416	0.720/12.225
**i5 -**	**i9**	0.753/2.527	0.798/1.824	0.860/2.620	0.678/6.173	0.696/7.806	0.729/10.431
**i6 -**	**i10**	0.775/1.801	0.809/1.460	0.870/1.446	0.719/4.010	0.742/5.383	0.759/7.283
**i7 -**	**i11**	0.793/1.422	0.816/1.236	0.873/0.859	0.765/2.342	0.782/3.810	0.800/3.257
**i8 -**	**i12**	0.812/1.144	0.821/1.111	0.876/0.605	0.809/1.501	0.812/3.008	0.827/1.636
**i9 -**	**i13**	0.828/0.941	0.826/1.162	0.877/0.506	0.841/1.136	0.829/2.682	0.842/1.053
**i10-**	**i14**	0.839/0.870	0.824/1.320	**0.878/0.498**	**0.850/0.963**	**0.836/2.853**	0.850/0.923
**i11-**	**i15**	0.840/0.919	0.809/1.737	0.878/0.601	0.849/1.038	0.833/3.527	0.855/0.944
**e1 -**	**e5**	0.489/2.270	0.765/0.541	0.878/52.799	0.536/1.005	0.765/0.121	0.865/0.145
**e2 -**	**e6**	0.515/1.804	0.759/0.445	0.881/0.437	0.569/0.850	0.792/0.109	0.881/0.164
**e3 -**	**e7**	0.526/1.325	0.752/0.340	0.880/0.386	0.579/0.706	0.816/0.095	0.881/0.160
**e4 -**	**e8**	0.540/0.987	0.739/0.274	0.878/0.349	0.589/0.575	0.836/0.083	0.881/0.166
**e5 -**	**e9**	0.563/0.729	0.725/0.243	0.875/0.307	0.604/0.446	0.854/0.078	0.881/0.179
**e6 -**	**e10**	0.583/0.568	0.726/0.205	0.876/0.240	0.607/0.371	0.866/0.069	0.881/0.180
**e7 -**	**e11**	0.597/0.507	0.747/0.171	0.876/0.171	0.612/0.297	0.874/0.061	**0.881/0.168**
**e8 -**	**e12**	0.603/0.486	0.776/0.138	0.874/0.133	0.619/0.252	0.878/0.053	0.880/0.161
**e9 -**	**e13**	0.601/0.474	0.808/0.110	0.869/0.114	**0.625/0.198**	0.880/0.050	0.879/0.164
**e10-**	**e14**	0.592/0.438	0.839/0.084	0.858/0.105	0.623/0.157	**0.883/0.049**	0.875/0.162
**e11-**	**e15**	0.577/0.412	0.856/0.072	0.839/0.103	0.608/0.140	0.882/0.055	0.865/0.160
